# Multipoint Pacing Versus Cardiac Resynchronization Therapy in Heart Failure: A Systematic Review and Meta‐Analysis

**DOI:** 10.1111/pace.70259

**Published:** 2026-04-18

**Authors:** Fahad Muhammad, Mohammad AlMeer, Eyad Jamileh, Ahmed Elmewafy, Ibrahim Antoun

**Affiliations:** ^1^ Faculty of Medicine and Dentistry Queen Mary University of London London UK; ^2^ School of Medicine University of Central Lancashire, England Preston UK; ^3^ Royal Blackburn Teaching Hospital East Lancashire Hospitals Trust, England Blackburn UK; ^4^ School of Medicine Queen's University Belfast Northern Ireland Belfast UK; ^5^ School of Medicine University of Sheffield, England Sheffield UK; ^6^ Department of Cardiology University Hospitals of Leicester NHS Trust Glenfield Hospital Leicester UK; ^7^ Department of Cardiovascular Sciences Clinical Science Wing University of Leicester Glenfield Hospital Leicester UK

**Keywords:** conventional cardiac resynchronization, heart failure, multipoint pacing, systematic review

## Abstract

**Introduction:**

Multipoint pacing (MPP) delivers sequential stimuli from multiple left‐ventricular electrodes, potentially improving cardiac resynchronization therapy (CRT) response versus conventional biventricular pacing (BiV). We performed an updated systematic review and meta‐analysis to synthesize contemporary evidence.

**Methods:**

Following PRISMA 2020 (PROSPERO CRD420261293273), MEDLINE, EMBASE, and CENTRAL were searched to January 2026 for comparative studies of MPP versus conventional BiV in adults receiving CRT. Primary outcomes were all‐cause mortality and heart failure (HF)‐related hospitalization. Secondary outcomes included echocardiographic response, NYHA class improvement, and absolute change in left‐ventricular ejection fraction (LVEF). Random‐effects models produced pooled odds ratios (OR) or mean differences (MD).

**Results:**

Eight studies (*n* = 2430; 1190 MPP, 1240 BiV), including five randomized and three observational studies, were analyzed. All‐cause mortality showed no significant difference between groups (OR = 1.46, 95% CI 0.76–2.80; *p* = 0.25; *I* ^2^ = 0%). HF‐related hospitalization was significantly reduced with MPP in the largest trial (5.4% vs. 8.9%; *p* = 0.015), corresponding to a 39% relative risk reduction. MPP was associated with significantly higher echocardiographic response (OR = 0.43, 95% CI 0.29–0.64; *p* < 0.0001; *I* ^2^ = 0%), greater NYHA class improvement (OR = 0.38, 95% CI 0.20–0.73; *p* = 0.004; *I* ^2^ = 3%), and greater absolute LVEF (MD = −4.67, 95% CI −6.70 to −2.64; *p* < 0.00001; *I* ^2^ = 0%).

**Conclusions:**

Compared with conventional CRT, MPP was associated with improved functional and echocardiographic outcomes and reduced HF hospitalization, without a demonstrated mortality benefit. Larger prospective studies with longer follow‐up are required to assess long‐term prognostic effects.

**Abbreviations**
BiV: biventricular pacing
CI: confidence interval
CENTRAL: Cochrane Central Register of Controlled Trials
CRT: cardiac resynchronization therapy
HF: heart failure
LBBB: left bundle branch block
LV: left ventricle/left ventricular
LVEF: left ventricular ejection fraction
MD: mean difference
MPP: multipoint pacing
NYHA: New York Heart Association
OR: odds ratio
PRISMA: Preferred Reporting Items for Systematic Reviews and Meta‐Analyses
RCT: randomized controlled trial
RoB 2: Risk of Bias 2 tool
ROBINS‐I: Risk of Bias in Non‐randomized Studies of Interventions
SMD: standardized mean difference

## 1 Introduction

Conventional cardiac resynchronization therapy (CRT) relies on biventricular pacing (BiV) and is an established treatment for patients with heart failure (HF) and electrical dyssynchrony, mostly characterized by a left bundle branch block (LBBB) [1]. However, 20%–30% of patients fail to demonstrate a meaningful clinical or echocardiographic response following CRT implantation, and these non‐responders experience higher rates of HF hospitalization and mortality compared with responders [2, 3].

CRT non‐response is associated with multiple well‐established contributing factors, including suboptimal left ventricular (LV) lead position, myocardial scar, non‐LBBB conduction patterns, and programming. Impaired LV activation related to pacing lead position can be a modifiable mechanism, particularly in the presence of myocardial scar or heterogeneous conduction delay. Multipoint pacing (MPP) represents an evolution of conventional CRT, in which multiple LV pacing stimuli are delivered via a single quadripolar coronary sinus lead, aiming to improve electrical resynchronization by recruiting a broader myocardial activation wavefront [4, 5]. Early mechanistic and clinical studies demonstrated favorable effects of MPP on acute hemodynamic parameters and short‐term indices of dyssynchrony [5, 6, 7]. Nevertheless, adoption of MPP has been limited by increased programming complexity, reduced device battery longevity, and uncertainty regarding incremental clinical benefit over standard BiV [3, 8].

Mehta et al. 2021 previously conducted a meta‐analysis concluding an evident echocardiographic benefit with MPP; the study was largely driven by non‐randomized studies [9]. Since then, three randomized controlled trials (RCTs) have been published, including the MORE‐CRT trial, the largest study to date evaluating the clinical benefit of MPP [10]. Therefore, an updated systematic review and meta‐analysis is warranted to synthesize contemporary evidence and compare the efficacy and safety of MPP with conventional CRT.

## 2 Methods

### 2.1 Study Design and Reporting Standards

This systematic review and meta‐analysis have been performed in line with the Preferred Reporting Items for Systematic Reviews and Meta‐Analyses (PRISMA) 2020 statement [11]. The review protocol was prospectively registered with PROSPERO (CRD420261293273) prior to formal data extraction [12]. Any deviations from the registered protocol will be documented and justified in the final manuscript. PRISMA checklist is included in the supplementary material.

### 2.2 Eligibility Criteria

We included randomized and non‐randomized comparative studies of adult patients (≥18 years) diagnosed with HF of any etiology undergoing CRT, in which MPP was delivered using a multipolar LV lead as part of a CRT system. Eligible studies were required to include a comparator group receiving conventional BiV CRT or to report outcomes under baseline CRT settings prior to activation of MPP. Studies were required to report at least one clinically relevant endpoint, including all‐cause mortality, HF‐related hospitalizations, cardiovascular mortality, device‐ or procedure‐related adverse events, or quality of life measured using a validated tool.

We excluded pediatric studies, single‐arm studies without a comparator group, case reports, case series, conference abstracts, editorials, and review articles. Studies were also excluded if MPP could not be clearly distinguished from other CRT programming strategies, if participants undergoing CRT could not be separated from other cardiac populations, or if relevant outcome data were not reported independently.

### 2.3 Outcomes

The primary outcomes assessed were all‐cause mortality and HF‐related hospitalizations during follow‐up. Secondary outcomes included cardiovascular mortality, device‐ or procedure‐related adverse events (including lead‐related complications, infection, and pacing‐related complications where reported), and quality of life, assessed using any validated patient‐reported outcome measure. However, none of the included studies reported sufficient extractable data for cardiovascular mortality, device‐related adverse events, or quality of life outcomes; therefore, these outcomes could not be synthesized in the present analysis.

### 2.4 Literature Search

Systematic literature searches were performed in MEDLINE (via PubMed), EMBASE, and the Cochrane Central Register of Controlled Trials (CENTRAL) from database inception to January 2026. The full search strategies are presented in the supplementary material. The reference lists of included studies and relevant systematic reviews were hand‐searched to identify further eligible articles.

### 2.5 Study Selection

Two independent reviewers (F.M., E.J.) screened titles and abstracts and assessed the full texts against the inclusion criteria. Disagreements were resolved by discussion or arbitration with a third reviewer (A.E.).

### 2.6 Data Extraction

Data extraction was completed independently by two reviewers using a standardized, pre‐piloted Excel extraction form developed in accordance with Cochrane data collection guidance. Extracted data included study design, sample size, baseline participant characteristics, HF etiology and severity, electrocardiographic parameters (e.g., QRS duration where available), CRT indication, device, and lead characteristics, MPP programming strategy, comparator pacing configuration, follow‐up duration, and all prespecified clinical and safety outcomes.

### 2.7 Risk of Bias

Methodological quality and risk of bias were evaluated independently by two reviewers. RCTs were assessed using the Cochrane Risk of Bias 2 (RoB 2) Tool, which evaluated the following domains: randomization process, deviations in intervention delivery, missing outcome data, outcome management, and selective reporting. Based on these criteria, each RCT was categorized as having low, some concerns, or high risk of bias [13]. Non‐randomized comparative studies were appraised using the Risk of Bias in Non‐randomized Studies—of Interventions (ROBINS‐I) tool, which evaluated the following domains: confounding, participant selection, classification of interventions, deviations from intended interventions, missing data, outcome measurement, and selection of the reported result [14]. Domain‐level and overall risk of bias were subsequently categorized as low, moderate, serious, or critical. Any discrepancies between reviewers were resolved through discussion, with arbitration by a third reviewer when necessary.

### 2.8 Data Synthesis

Where at least two studies reported outcomes that were sufficiently comparable, results were combined using meta‐analysis in Review Manager (RevMan) version 5.3. Continuous variables were synthesized using either mean differences (MD) or standardized mean differences (SMD), each presented with 95% confidence intervals (CI). For dichotomous endpoints, pooled estimates were calculated as odds ratios (OR) with 95% CI. A random‐effects model was selected to account for expected clinical and methodological variability between studies. Statistical heterogeneity was evaluated using the *χ* ^2^ test and quantified with the *I* ^2^ statistic; thresholds were interpreted as low (0%–25%), moderate (25%–75%), and substantial (75%–100%). To standardize reporting, conventional CRT was consistently assigned as the control group. Therefore, a result favoring MPP is represented by an OR > 1 for adverse outcomes (e.g., mortality), and an OR < 1 or MD < 0 for positive outcomes (e.g., LVEF improvement). Where meta‐analysis was not appropriate due to insufficient data or heterogeneity in reporting, findings were summarized descriptively, emphasizing the direction and consistency of effects across studies. Planned subgroup analyses included comparisons according to study design (randomized vs. observational studies) and differences in study populations. However, the limited number of studies contributing to each outcome prevented robust subgroup or sensitivity analyses from being performed.

## 3 Results

### 3.1 Literature Search Results

**FIGURE 1:**
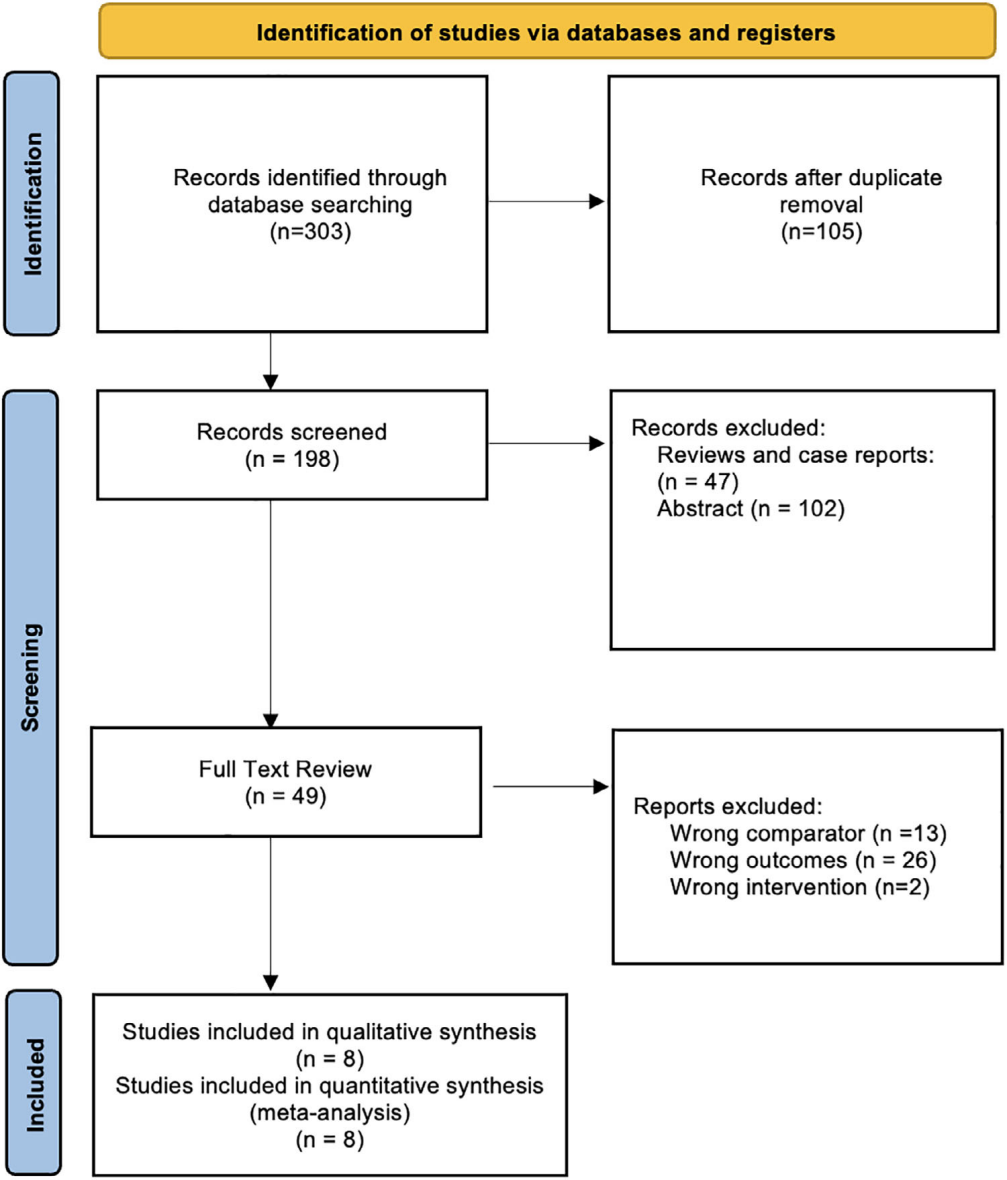
PRISMA Flowchart. [Colour figure can be viewed at wileyonlinelibrary.com]

Our search strategy retrieved 303 studies. After thoroughly screening the retrieved articles, the authors identified eight studies that met the eligibility criteria (Figure 1) [10, 15, 16, 17, 18, 19, 20, 21].

### 3.2 Description of Studies

Eight studies were included in the final analysis, totaling 2430 patients. Of these, 1190 patients received MPP and 1240 received CRT. Table 1 summarizes the baseline characteristics of the included studies. Five studies were RCTs, and three were observational studies. All studies evaluated patients with HF with reduced ejection fraction undergoing CRT implantation, comparing MPP with conventional BiV. Table 2 summarizes the baseline participant characteristics across studies.

**TABLE 1:** Baseline characteristics of the included studies.

Author (year)	Age (years)		Male (%)		Ischemic etiology (%)		NYHA functional class (I/II/III/IV)		LVEF (%) ± SD		QRSd (ms) ± SD		QRSm (% LBBB)	
	CRT	MPP	CRT	MPP	CRT	MPP	CRT	MPP	CRT	MPP	CRT	MPP	CRT	MPP
Papone et al. (2015) [15]	67 ± 8	66 ± 8	73	86	36	55	0/0/100/0	0/0/100/0	30 ± 6	28 ± 5	151 ± 17	153 ± 16	59	55
Zanon et al. (2016) [16]	69.7 ± 10.4	67.4 ± 12.5	68.5	80	50	55	1.9/20.4/68.5/9.3	0/15/80/5	30.4 ± 6.3	27.2 ± 4.3	NR	NR	61.1	65.0
Forleo et al. (2016) [17]	71 ± 10	69 ± 11	79	81	47	41	0/45/53/2	0/30/68/2	28.1 ± 6.0	28.2 ± 5.9	157 ± 23	164 ± 3	78	71
Niazi et al. (2017) [18]	68 ± 10	67 ± 10	66.1	63.7	48.9	47.8	6.1/32.8/58.3/2.8	12.4/25.9/56.2/5.5	NR	NR	154 ± 20	158 ± 24	77.2	73.4
Marques et al. (2021) [19]	66.5 ± 11.6	64.5 ± 10.2	60	54.2	16	20.8	0/68/32/0	0/70.8/29.2/0	29.6 ± 6.9	28.7 ± 6.9	164 [IQR: 150, 174]	170 [IQR: 157, 183]	64.0	62.5
Almusaad et al. (2021) [20]	59.6 [IQR: 52.1, 67.0]	62.1 [IQR: 52.3, 69.4]	69.5	65.8	30.4	35.6	0/23.2/72.5/4.3	0/15.1/78.1/6.8	25.7 [IQR: 21.7, 32.5]	26.0 [IQR: 21.1, 33.6]	158.0 [IQR: 150.0, 170.0]	160.0 [IQR: 150.0, 172.3]	100	100
Passafaro et al. (2024) [21]	NR	NR	NR	NR	NR	NR	NR	NR	30.3 ± 3.9	26.1 ± 6.3	155	159	NR	NR
Leclercq et al. (2025) [10]	68 ± 11	68 ± 10	75.5	78.3	49.5	52.5	0/47.9/49.8/2.0	0/50.1/47.2/2.4	29 ± 8	29 ± 8	155 ± 24	155 ± 26	63.6	62.8

**TABLE 2:** Baseline characteristics of participants included in each study.

Author (year)	Journal	Region	Study design	Single or multi‐center	Population	Comparison	CRT device model	LV lead type	MPP programming strategy	Recruitment period (months)	Follow‐up duration	Total sample (n)	MPP group (n)	CRT group (n)
Papone et al. (2015) [15]	Heart Rhythm	United States	RCT	Single	CRT implant according to ESC/EHRA guidelines	MPP vs. Conventional BiV CRT	Unify Quadra MP/Quadra Assura MP | CRT‐D	Quartet Quadripolar LV lead	Sequential (PV loop‐guided)	8	12 months (post‐implant)	42^a^	21	21
Zanon et al. (2016) [16]	Heart Rhythm	Italy	Observational Study	Single	CRT implant according to ESC/EHRA guidelines	MPP vs. Conventional BiV CRT	Quadra Assura MP | CRT‐D	Quartet Quadripolar LV lead	Sequential (Electrical delay‐guided)	15 (STD); 16 (MPP)	12 months (post‐implant)	74	20	54
Forleo et al. (2017) [17]	Europace	Italy	Observational Study	Multi‐center	Patients undergoing CRT‐D implantation | De novo or upgrade	MPP vs. Conventional BiV CRT	Unify Quadra MP/Quadra Assura MP | CRT‐D	Quartet Quadripolar LV lead	Not specified	22	6 months (post‐implant)	232	94	138
Niazi et al. (2017) [18]	JACC: Clinical Electrophysiology	United States	RCT	Multi‐center	Patients undergoing CRT‐D implantation | De novo or upgrade | AF excluded	MPP vs. Continued BiV CRT (after 3‐month BiV CRT run‐in)	Quadra MP | CRT‐D	Quartet Quadripolar LV lead	Sequential (MPP‐AS 5 ms; MPP‐Other variable)	14	6 months (post 3 month run‐in)	381	201	180
Marques et al. (2021) [19]	Pacing and Clinical Electrophysiology	Portugal	RCT	Single	Post‐CRT patients | Responders only | AF excluded	MPP vs. Continued BiV CRT (after 6‐month BiV CRT run‐in)	Quadra Allure MP (CRT‐P)/Quadra Assura MP (CRT‐D)	Quartet Quadripolar LV lead	Sequential (5 ms delay)	24	6 months (post 6 month run‐in)	49	24^b^	25
Almusaad et al. (2021) [20]	Journal of Interventional Cardiac Electrophysiology	Middle East	RCT	Multi‐center	Patients undergoing CRT‐D implantation | De novo | LBBB only | AF excluded	MPP vs. Conventional BiV CRT	Unify Quadra MP/Quadra Assura MP | CRT‐D	Quartet Quadripolar LV lead	Sequential (Automated VectSelect)	NR	6 months (post‐implant)	142	73	69
Passafaro et al. (2024) [21]	The American Journal of Cardiology	Italy	Observational Study	Multi‐center	CRT‐D implantation | ESC Class I indication	MPP vs. Conventional BiV CRT	Quadra Assura MP | CRT‐D	Quartet Quadripolar LV lead	Not specified	39	36 months (post‐implant)	51	34	17
Leclercq et al. (2025) [10]	Europace	France	RCT	Multi‐center	Post‐CRT patients | Non‐responders only | AF excluded	MPP vs. Continued BiV CRT (after 6‐month BiV CRT run‐in)	Quadra CRT	Quartet Quadripolar LV lead	Sequential (Physician‐directed programming)	NR	5 ± 1 months (post 6 month run‐in)	1421	722	699

*Note*: All CRT Devices and LV leads were manufactured by Abbott (previously St. Jude Medical). (a) Pappone et al. (2015) had 44 patients randomized, MPP: 22 randomized, 21 analyzed at 3 months, 19 analyzed at 12 months; CRT: 22 randomized, 22 analyzed at 3 months, 21 analyzed at 12 months. (b) Marques et al. (2021) had 2/24 MPP patients crossed over to BiV due to tolerability.

## 4 Primary Outcomes

### 4.1 All‐Cause Mortality

**FIGURE 2:**
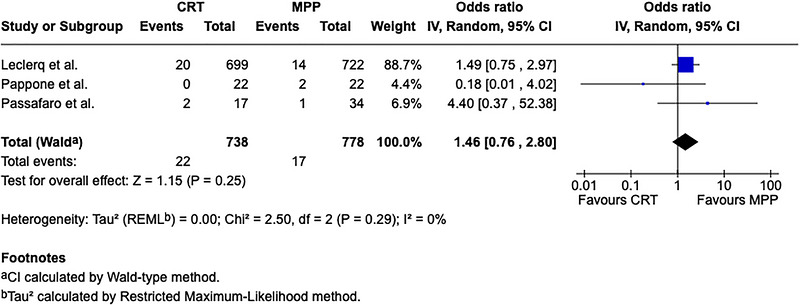
Forest plot of all‐cause mortality comparing conventional CRT and MPP. [Colour figure can be viewed at wileyonlinelibrary.com]

No statistically significant difference in all‐cause mortality was observed between the MPP and conventional CRT (OR = 1.46, 95% CI 0.76–2.80; *p* = 0.25). Although the point estimate numerically favored MPP, the confidence interval crossed the line of no effect. Statistical heterogeneity among the included studies was absent (*I* ^2^ = 0%, *p* = 0.29). This is shown in Figure 2.

### 4.2 HF‐Related Hospitalization

Only one study (Leclercq et al.) reported estimable data for HF‐related hospitalization; therefore, a pooled meta‐analysis for this endpoint was not feasible [10]. In this study, HF‐related hospitalizations occurred in 5.4% (39/722) of patients in the MPP group compared with 8.9% (62/699) in the conventional CRT group, representing a 39% relative risk reduction (*p* = 0.0154).

## 5 Secondary Outcomes

### 5.1 Echocardiographic Endpoints

**FIGURE 3:**
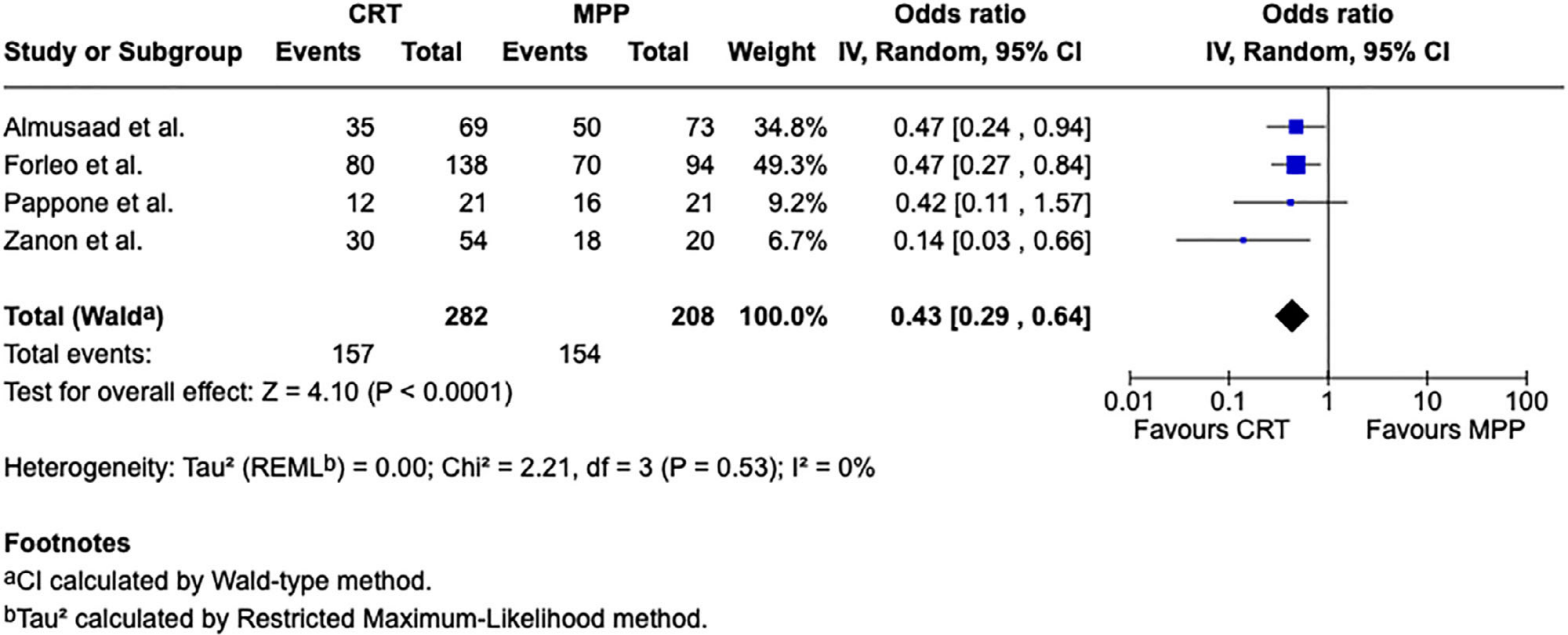
Forest plot of favorable echocardiographic response comparing conventional CRT and MPP. Forleo et al. (2016) counts were derived from reported percentages and the number of patients with available follow‐up data. [Colour figure can be viewed at wileyonlinelibrary.com]

MPP was associated with a significantly higher rate of favorable echocardiographic response compared with conventional CRT (OR = 0.43, 95% CI 0.29–0.64; *p* < 0.0001). Statistical heterogeneity among the included studies was absent (*I* ^2^ = 0%, *p* = 0.53). This is shown in Figure 3.

### 5.2 New York Heart Association Class Improvement

**FIGURE 4:**
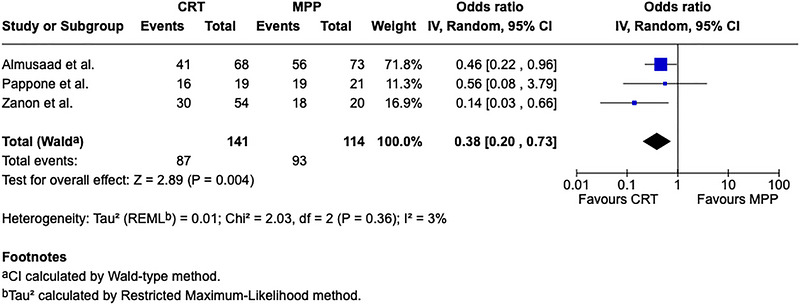
Forest plot of NYHA functional class improvement comparing conventional CRT and MPP. [Colour figure can be viewed at wileyonlinelibrary.com]

A statistically significant difference in New York Heart Association (NYHA) functional class improvement was observed between the MPP and conventional CRT, with a higher rate of improvement in the MPP group (OR = 0.38, 95% CI 0.20–0.73; *p* = 0.004). Heterogeneity among the included studies was low (*I* ^2^ = 3%, *p* = 0.36). This is shown in Figure 4.

### 5.3 Absolute Change in Left Ventricular Ejection Fraction (LVEF)

**FIGURE 5:**
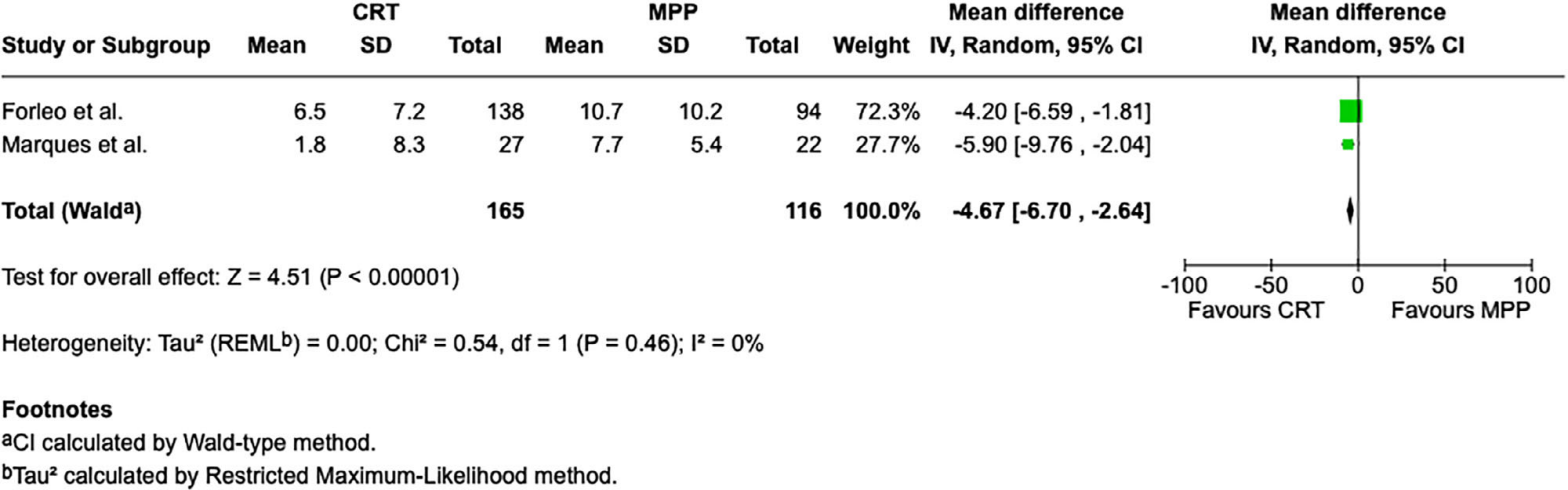
Forest plot of absolute change in LVEF comparing conventional CRT and MPP. Forleo et al. (2016) measures from baseline to 6 months, Marques et al. (2021) measures from 6 months post‐CRT run‐in. [Colour figure can be viewed at wileyonlinelibrary.com]

A statistically significant difference in the absolute change in LVEF between MPP and conventional CRT was observed, with greater improvement in the MPP group (MD = −4.67, 95% CI −6.70 to −2.64; *p* < 0.00001). No statistical heterogeneity was observed (*I* ^2^ = 0%, *p* = 0.46). This is shown in Figure 5.

### 5.4 Other Prespecified Secondary Outcomes

Cardiovascular mortality, device‐related adverse events, and quality of life were prespecified secondary outcomes. However, none of the included studies reported sufficient extractable data for these outcomes, and therefore quantitative synthesis was not possible.

### 5.5 Risk of Bias Assessment

**FIGURE 6:**
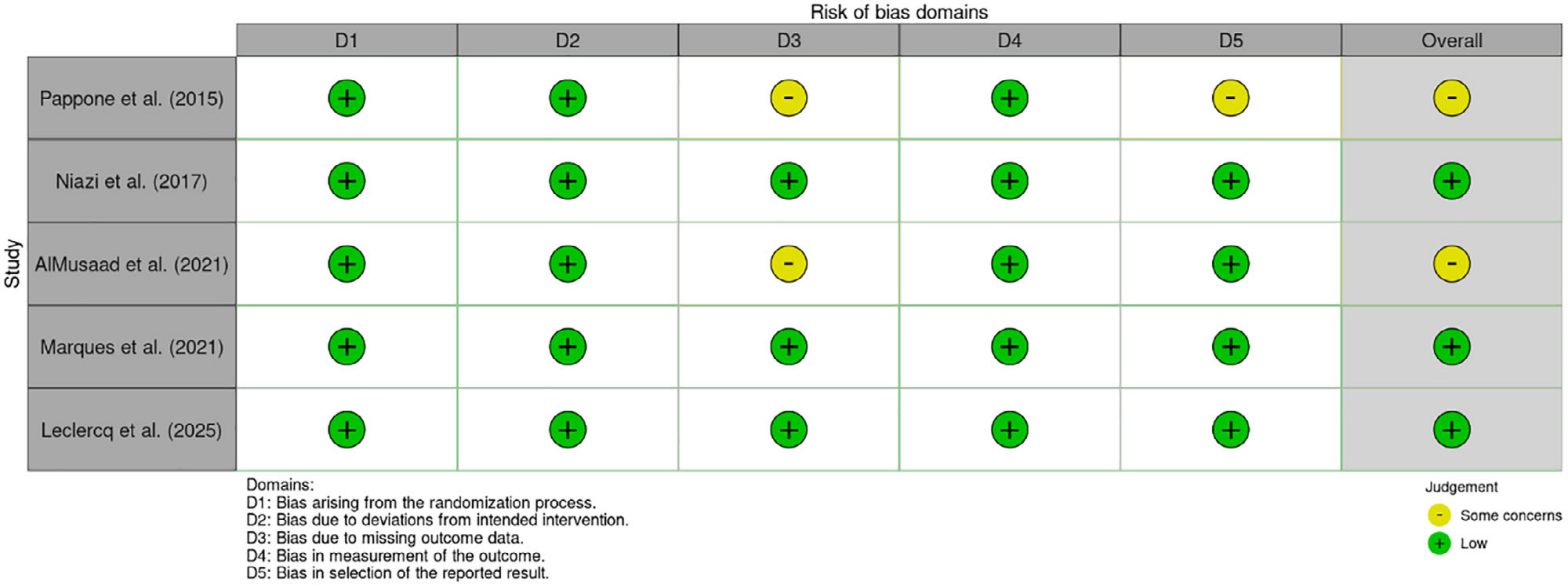
Risk of bias summary for each individual study using the RoB 2 tool. [Colour figure can be viewed at wileyonlinelibrary.com]

**FIGURE 7:**
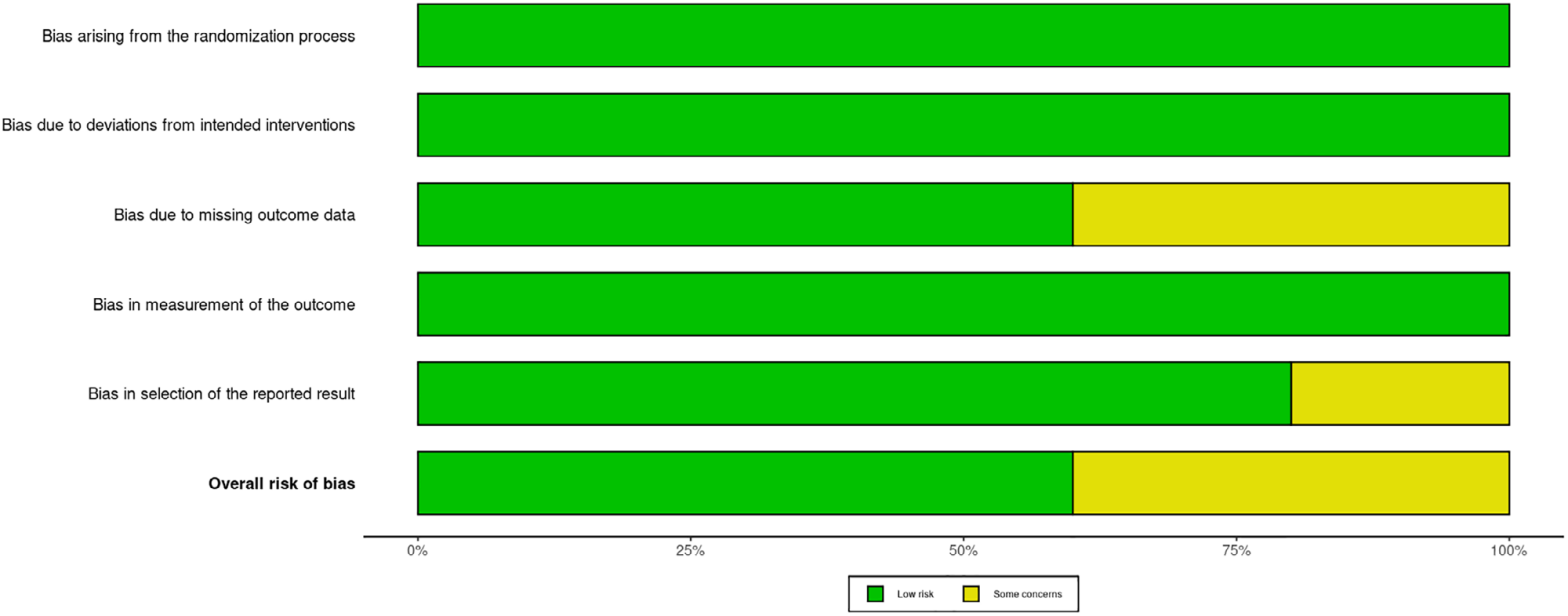
Proportion of risk of bias judgements across all included studies using the RoB 2 tool. [Colour figure can be viewed at wileyonlinelibrary.com]

**FIGURE 8:**
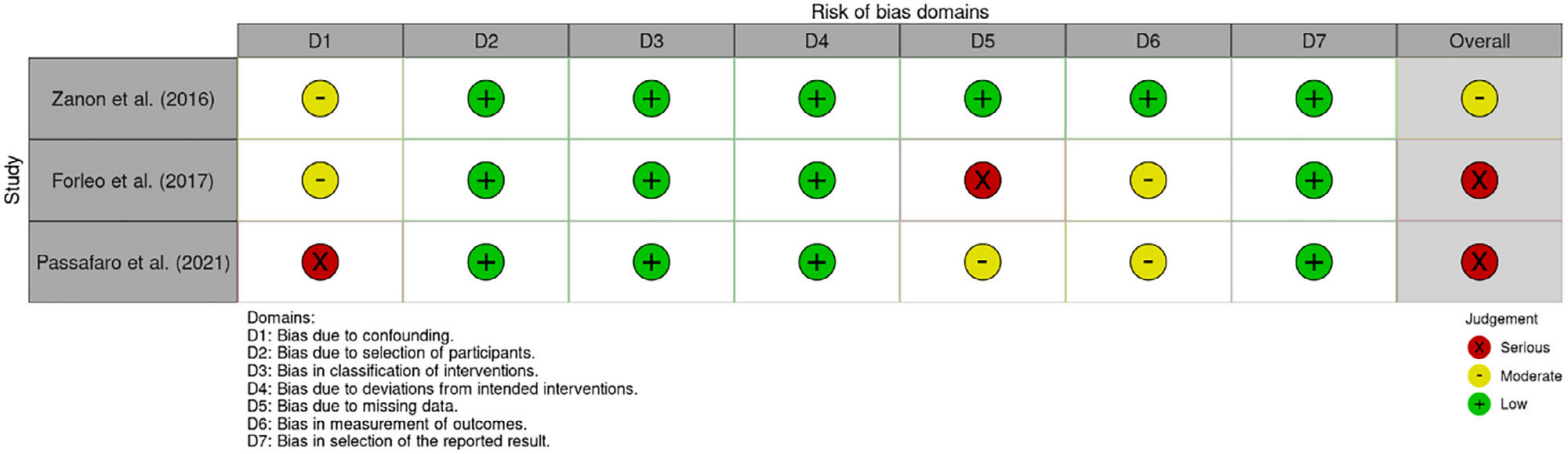
Risk of bias summary for each individual study using the ROBINS‐I tool. [Colour figure can be viewed at wileyonlinelibrary.com]

**FIGURE 9:**
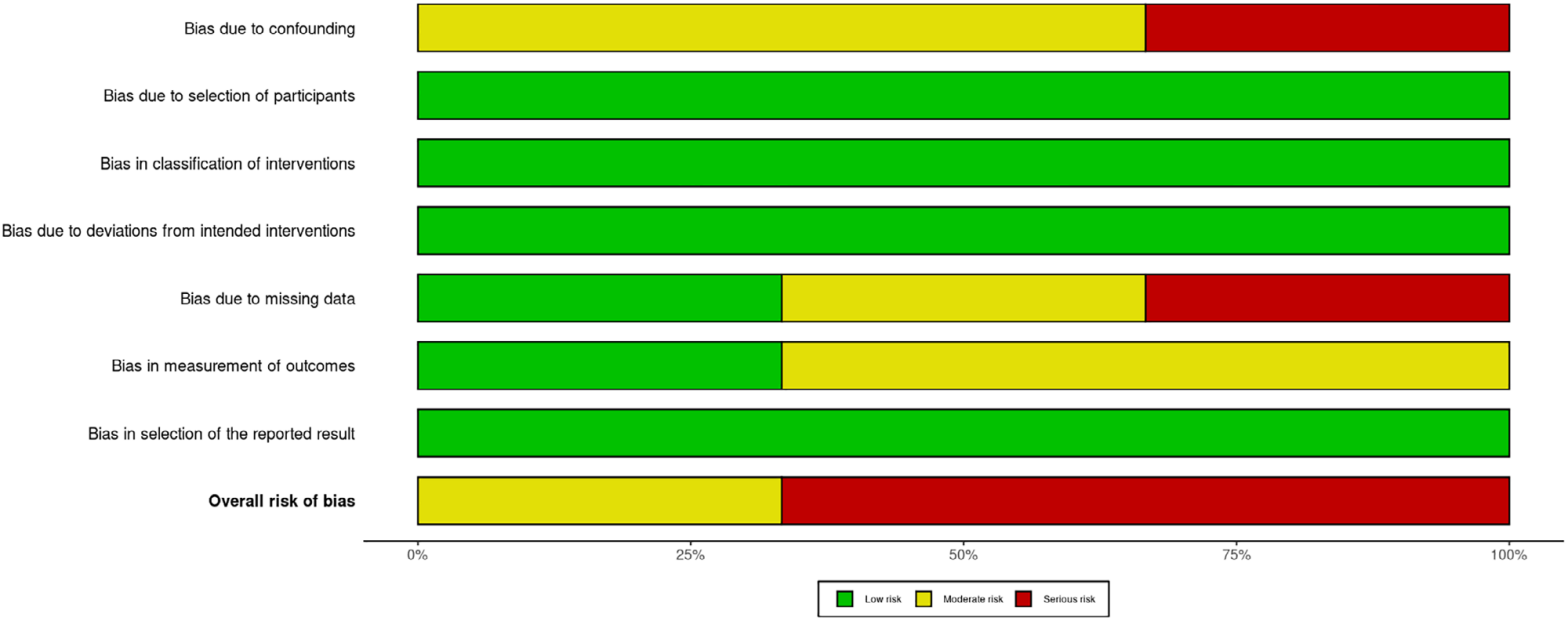
Proportion of risk of bias judgements across all included studies using the ROBINS‐I tool. [Colour figure can be viewed at wileyonlinelibrary.com]

Risk of bias in RCTs was assessed using the Cochrane RoB 2 tool, shown graphically in Figures 6 and 7. Observational studies were evaluated using the ROBINS‐I tool, shown graphically in Figures 7 and 8 [13, 14]. The proportion of risk of bias judgements across all included studies using the ROBINS‐I tool are shown graphically in Figure 9. Five RCTs and three observational studies were included.

The included RCTs demonstrated high methodological quality. The risk of bias arising from the randomization process, intervention deviations, and outcome measurement was low across all trials. Missing outcome data were generally low risk, though Almusaad et al. and Pappone et al. raised concerns about sample attrition [15, 20]. Selective reporting was low risk across all studies, except for Pappone et al., which raised some concerns [15]. Consequently, Niazi et al., Marques et al., and Leclercq et al. were categorized as having an overall low risk of bias, whilst Almusaad et al. and Pappone et al. raised some overall concerns [10, 15, 18, 19, 20].

The three observational studies were judged to range from a moderate to serious overall risk of bias using the ROBINS‐I tool. Notably, no study was assessed as being at a critical risk of bias, thereby meeting the methodological threshold for inclusion in the synthesis. Overall, while the evidence base provides valuable real‐world clinical data, it remains fundamentally limited by significant baseline confounding and notable attrition across two cohorts.

## 6 Discussion

This updated systematic review and meta‐analysis of eight studies, including 2430 patients, provides a contemporary evaluation of MPP compared with BiV in CRT. Three principal findings have emerged.

First, MPP was consistently associated with improvements in surrogate measures of CRT response. Compared with conventional CRT, MPP significantly increased the likelihood of echocardiographic response and improvement in the NYHA functional class and was associated with a greater absolute increase in LV ejection fraction (MD, 4.67%). These findings, observed across multiple included trials, indicate that MPP is associated with enhanced reverse remodeling and symptomatic improvement [15, 17, 19, 20].

Second, MPP was associated with reduced heart‐failure‐related hospitalization. This finding was primarily informed by the MORE‐CRT MPP trial, which demonstrated a significant 39% relative risk reduction in HF‐related hospitalizations [10]. However, this trial specifically enrolled patients who had failed to demonstrate reverse remodeling after conventional CRT, representing a population of CRT non‐responders. Therefore, while this result suggests a potential morbidity benefit of MPP in selected patients, it should be interpreted cautiously. The current evidence for this endpoint is derived from a single study and lacks confirmation across other trials, limiting the generalizability of this finding to the broader population of patients receiving CRT.

Third, no statistically significant reduction in all‐cause mortality was observed. Although the point estimate numerically favored MPP (OR 1.46; 95% CI 0.76–2.80), the confidence interval was wide and crossed unity, indicating substantial uncertainty. Therefore, current evidence does not support a survival advantage of MPP.

Our findings refine the conclusions of the most recent major meta‐analysis by Mehta et al., which demonstrated echocardiographic improvement but insufficient evidence for clinical outcome benefit [9]. The inclusion of contemporary studies, particularly the long‐term follow‐up from Passafaro et al. (2024) and the large randomized MORE‐CRT MPP trial, provides additional insight into clinical outcomes [10, 21].

Importantly, the MORE‐CRT MPP trial randomized patients who had failed to demonstrate reverse remodeling after 6 months of standard CRT [10]. In this clinical context, the 39% reduction in HF events observed in that trial provides a compelling signal of morbidity benefit for “non‐responders.” While our broader analysis demonstrated that functional and echocardiographic improvements are consistent across study designs, the specific reduction in hospitalizations remains informed by a single large cohort. Therefore, the current evidence suggests that MPP is a highly effective optimization strategy for selected patients, particularly those with suboptimal response to conventional CRT, rather than a mandatory universal replacement for conventional CRT.

The observed pattern of findings, namely, improvements in reverse remodeling and symptomatic status in the absence of a demonstrable mortality benefit, mirrors the historical trajectory of evidence underpinning CRT itself. Structural and functional improvements typically precede survival divergence, which often requires larger populations and longer follow‐up periods for detection. Given that most included studies had follow‐up durations of 6–12 months, the absence of a mortality difference likely reflects insufficient statistical power, short follow‐up duration, and the low number of observed endpoint events across included studies. In the pooled dataset, only 39 total deaths were reported (22 in the CRT group and 17 in the MPP group), which substantially limits the ability of meta‐analysis to detect meaningful differences in survival.

The clinical findings are biologically plausible; conventional CRT relies on a single LV pacing site and may not adequately overcome electrical dyssynchrony in the presence of conduction delay heterogeneity or myocardial scar [4]. MPP delivers sequential stimulation from two electrodes on the same quadripolar lead, potentially recruiting a larger myocardial volume and shortening the activation time across the left ventricle [5].

Experimental and hemodynamic studies provide a supportive context. Prior work by Niazi et al. (2017) demonstrated that maximizing the spatial distance between the LV pacing electrodes (>30 mm) correlates with improved clinical response, suggesting that broader recruitment of the LV myocardium is a key determinant of CRT efficacy [18]. Similarly, both invasive and non‐invasive hemodynamic optimization studies have shown improved contractility and stroke volume with MPP compared with single‐site pacing [21, 22]. Therefore, the magnitude of LVEF improvement observed in our analysis is consistent with a true physiological effect rather than measurement variability.

Improved ventricular synchrony reduces filling pressures and pulmonary congestion, providing a mechanistic explanation for the observed reduction in HF hospitalizations [23]. However, the absence of a mortality effect suggests that MPP primarily modifies the HF status rather than altering the underlying disease trajectory.

Approximately 20%–30% of patients receiving CRT fail to demonstrate an adequate clinical response [2]. Our findings suggest that MPP is best conceptualized as an optimization strategy rather than a default pacing configuration.

Routine activation of the MPP in all de novo implants is not supported by current evidence. MPP requires the delivery of 2 LV pacing impulses per cardiac cycle and is associated with reduced device longevity; prior analyses estimate a 15%–20% reduction in battery life [8]. In the absence of proven survival benefit, earlier generator replacement and associated procedural risk limit the justification for universal programming [16, 24].

Instead, evidence supports selective use. Patients with persistent symptoms, lack of reverse remodeling, or suspected electrical or scar‐related dyssynchrony after CRT implantation represent the population most likely to benefit from this therapy [10]. In such cases, potential reductions in hospitalization and improvement in functional status may outweigh the cost of battery longevity [10, 16]. Emerging automated vector‐selection algorithms may also facilitate implementation without complex manual optimization [20].

## 7 Limitations

This study has several limitations. The number of included studies remains modest, and both randomized and observational designs were pooled, introducing potential bias and heterogeneity. The HF hospitalization outcome was reported by only a single large trial and therefore lacks confirmation across multiple independent datasets [10]. The programming strategies varied substantially across studies, resulting in inconsistent myocardial resynchronization. In addition, while one study included a 36‐month follow‐up, the majority reported follow‐up durations of only 6 to 12 months. This relatively short observation period limits the ability to detect meaningful differences in all‐cause mortality, which typically requires longer follow‐up in the context of a chronic progressive condition such as HF. Finally, the small number of included studies and inconsistent reporting of outcomes limited the feasibility of subgroup and sensitivity analyses, which may have provided further insight into differences according to study design or patient population.

## 8 Conclusion

This updated systematic review and meta‐analysis represent the largest contemporary synthesis of MPP in CRT. Our findings indicate that MPP is associated with significant improvements in echocardiographic response and functional status compared with conventional pacing. While data from a large randomized trial suggest a potential reduction in HF hospitalizations, this finding is currently limited to a single study and lacks confirmation in the broader evidence base. Furthermore, these functional and morbidity signals have not yet translated into a statistically significant improvement in all‐cause mortality. Consequently, MPP should be conceptualized as a physiological optimization tool rather than a proven survival therapy. Routine prophylactic activation is not currently supported, given the associated reduction in device longevity; instead, MPP may be most appropriate for patients with persistent symptoms or a sub‐optimal response to conventional CRT. Future multicenter RCTs with extended follow‐up are essential to determine whether these observed improvements in reverse remodeling eventually yield a long‐term survival benefit. Research should also focus on standardizing programming protocols to identify patient phenotypes most likely to derive prognostic benefit.

### 8.1 Registration and Protocol

This systematic review was registered in the PROSPERO International Register of Systematic reviews, registration number: CRD420261293273. The protocol is available from https://www.crd.york.ac.uk/PROSPERO/view/CRD420261293273. No amendments were made to the protocol.

## Author Contributions

**Fahad Muhammad**: conceptualization, data curation, formal analysis, investigation, writing – original draft. **Mohammad AlMeer**: data curation, formal analysis, visualization, writing – original draft. **Eyad Jamileh**: investigation (secondary reviewer), validation, writing – original draft, writing – review & editing, final approval. **Ahmed Elmewafy**: writing – review & editing, final approval. **Ibrahim Antoun**: supervision, project administration, writing – review & editing, final approval.

## Ethics Statement

A Statement of Ethics is not applicable because this study is based exclusively on published literature.

## Conflicts of Interest

The authors have no conflicts of interest to declare.

## Data Availability

All data generated or analyzed during this study are included in this article. Further enquiries can be directed to the corresponding author.
